# Simple and Inexpensive Paper-Based Astrocyte Co-culture to Improve Survival of Low-Density Neuronal Networks

**DOI:** 10.3389/fnins.2018.00094

**Published:** 2018-02-27

**Authors:** Mathias J. Aebersold, Greta Thompson-Steckel, Adriane Joutang, Moritz Schneider, Conrad Burchert, Csaba Forró, Serge Weydert, Hana Han, János Vörös

**Affiliations:** Laboratory of Biosensors and Bioelectronics, Institute for Biomedical Engineering, ETH Zürich, Zurich, Switzerland

**Keywords:** neuron, astrocyte, co-culture, paper-based, low-density culture, cell viability, neurite length, network activity

## Abstract

Bottom-up neuroscience aims to engineer well-defined networks of neurons to investigate the functions of the brain. By reducing the complexity of the brain to achievable target questions, such *in vitro* bioassays better control experimental variables and can serve as a versatile tool for fundamental and pharmacological research. Astrocytes are a cell type critical to neuronal function, and the addition of astrocytes to neuron cultures can improve the quality of *in vitro* assays. Here, we present cellulose as an astrocyte culture substrate. Astrocytes cultured on the cellulose fiber matrix thrived and formed a dense 3D network. We devised a novel co-culture platform by suspending the easy-to-handle astrocytic paper cultures above neuronal networks of low densities typically needed for bottom-up neuroscience. There was significant improvement in neuronal viability after 5 days *in vitro* at densities ranging from 50,000 cells/cm^2^ down to isolated cells at 1,000 cells/cm^2^. Cultures exhibited spontaneous spiking even at the very low densities, with a significantly greater spike frequency per cell compared to control mono-cultures. Applying the co-culture platform to an engineered network of neurons on a patterned substrate resulted in significantly improved viability and almost doubled the density of live cells. Lastly, the shape of the cellulose substrate can easily be customized to a wide range of culture vessels, making the platform versatile for different applications that will further enable research in bottom-up neuroscience and drug development.

## Introduction

Picking apart the immeasurable number of parallel functions occurring within the brain remains an overwhelming challenge. A complementary approach to investigating the brain in its entirety is to develop *in vitro* cell-based assays designed to answer a specific research question. This minimizes the many confounding variables observed *in vivo*. The bottom-up neuroscience approach aims to engineer well-defined cellular micro-environments *in vitro* to probe the fundamental mechanisms of distinct neuronal populations (Aebersold et al., 2016). By reducing the complexity, *in vitro* bioassays can better control experimental variables and can provide significant value for fundamental research on how the nervous system develops and functions. In addition, bottom-up neuroscience methods are a robust, versatile tool for high throughput pharmacological research and development of drug targets against neurodevelopmental and neurodegenerative disorders (Jones et al., 2011; Choi et al., 2013; Bicker et al., 2014; Pamies et al., 2014; Kim et al., 2015; Terrasso et al., 2015, 2017; Fukushima et al., 2016; Sandström et al., 2017).

Intensified interest in functional micro-environments has led to a reconsideration of how to design cell culture systems to increase the physiological relevance of *in vitro* bioassays, as it is critical that simplicity is balanced with accuracy and precision. Standard cell culture techniques can be limited by poor cell viability especially at lower cell densities, despite the access to commercially available media formulations specialized for long-term culturing of different cell types. Particular to neuro-based assays, cells must often be cultured for 2 weeks or longer to achieve connected neuronal networks that exhibit spontaneous electrophysiological activity comparable to the developing nervous system *in vivo* (O'Donovan, 1999). While high density neuronal cultures tend to have acceptable cell survival rates and functional activity, lower density cultures would allow for the targeting and measuring of individual cells or neurites within a defined neuronal network. Single cell and small population analysis increases the precision of experimental cause and effect compared to the complexity of network functions both *in vivo* and in dense *in vitro* cultures. There is thus a need to both increase cell survival at lower cell densities and to provide a simplified, yet physiologically relevant, micro-environment for comparable cell response *in vitro*.

Efforts toward meaningful cell patterning have helped to increase the physiological relevance of the experimental culture compared to organized, 3D populations *in vivo* (Goubko and Cao, 2009; Roy et al., 2013; Matsusaki et al., 2014; Albers et al., 2015; Tomba and Villard, 2015; Aebersold et al., 2016; Alagapan et al., 2016; Honegger et al., 2016). Techniques exist both in 2D, with methods such as microcontact printing, and in 3D, with the development of novel 3D culture substrates (Birgersdotter et al., 2005; Huh et al., 2011; Edmondson et al., 2014; Knight and Przyborski, 2015; Ravi et al., 2015; Dermutz et al., 2017). Functionalizing culture substrates with extracellular matrix proteins and other key factors is necessary not only for basic cell adhesion and viability but also for creating versatile, defined environments.

Additional efforts have been focused on recreating the composition of the extracellular environment that the experimental culture is exposed to by developing specialized synthetic media (Brewer et al., 1993, 2008) and conditioned medium (Boehler et al., 2007; Fukushima et al., 2016), or by co-culturing methods with supporting cells either directly within the culture as a feeder layer (Wang and Cynader, 1999; Yang et al., 2005; Odawara et al., 2013) or physically separated (Kaech and Banker, 2006; Fath et al., 2009; Majumdar et al., 2011; Pyka et al., 2011; Geissler and Faissner, 2012; Jones et al., 2012; Shi et al., 2013; Gottschling et al., 2016). Co-culture techniques using compartments or inserts have also successfully increased cell viability by supplementing the extracellular micro-environment without perturbing the experimental culture (Pyka et al., 2011; Dinh et al., 2013; Ehret et al., 2015; Gottschling et al., 2016).

Astrocytic conditioned medium and astrocyte co-cultures are of particular value to *in vitro* neuronal cultures (Banker, 1980). Astrocytes have major roles in the development, support, and maintenance of the central nervous system, with functions including the secretion of growth factors, gliotransmitters, and extracellular matrix proteins, the recycling of neurotransmitters, and the regulation of ion concentrations that affect neurotransmission (Perea et al., 2009, 2014; Clarke and Barres, 2013; Allen, 2014; Chung et al., 2015; Khakh and Sofroniew, 2015). Various studies have aimed to test neuron-astrocyte interactions using *in vitro* co-culture systems. These have included testing for interactions during development, such as the astrocytic effect on differentiation of precursor cells and stem cells (Johnson et al., 2007; Tang et al., 2013; Ehret et al., 2015; Lischka et al., 2017; Xie et al., 2017; Schutte et al., 2018), synchronization of neuronal network activity (Kuijlaars et al., 2016), and number of formed synapses (Pyka et al., 2011; Jones et al., 2012; Shi et al., 2013), and also the effect during disease states, such as in ALS (Kunze et al., 2013), thymine deficiency (Park et al., 2001), oxidative stress (Kidambi et al., 2008), and general neurotoxicity (Anderl et al., 2009). While the relevance of co-cultures of neurons and astrocytes has become more evident as more and more functions of astrocytes are discovered, their cumulative effect on the survival of low-density networks is seldom addressed and remains unclear.

Cellulose filter paper has been demonstrated to be a mechanically stable material to use as a 3D culture substrate (Derda et al., 2009, 2011; Dermutz et al., 2017). The material is commercially available, inexpensive, biocompatible, bioinert, and ion and nutrient permeable (Akram et al., 2015). Various cell types, including primary neurons, readily adhere and integrate into the 3D porous fiber matrix, which can be functionalized with standard adhesion-promoting proteins (Derda et al., 2009, 2011; Dermutz et al., 2017). In addition, major advantages of using paper as a culture substrate are that the material is easy to handle and is compatible with standard sample preparation methods. It can also be structurally patterned via cutting, folding, rolling, and stacking, allowing for a customizable platform for versatile applications (Akram et al., 2015).

Here, we propose the use of cellulose filter paper for the construction of 3D primary astrocytic cultures. Because the paper substrate can easily be transferred between culture vessels, we also introduce the concept that it is an optimal co-culture platform. Astrocytes can develop separately from an experimental neuronal culture and then later be introduced by simply transferring the paper culture and suspending it over the experimental culture. With this indirect co-culture method, neuronal networks are exposed to critical secreted factors while remaining a distinguishable cell population. The supportive effect of co-culturing was assessed at a wide range of densities, from individual cells to well-connected networks, by quantifying changes in viability, neurite length and spiking activity.

## Materials and methods

### Cell culture

All experiments were performed with primary cells from cerebral cortices of E18 embryos of time-mated pregnant Wistar rats (Harlan Laboratories, Netherlands). Animal experiments were approved by the Cantonal Veterinary Office Zurich. The cortices were dissociated in a 37°C/5% CO_2_ incubator for 15 min in 5 mL of a filter-sterilized solution of 0.5 mg/mL papain (P4762, Sigma–Aldrich, Switzerland) and 15 Kunitz units/mL deoxyribonuclease I (D5025-15KU, Sigma–Aldrich) in PBS (10010015, Gibco, Thermo Fisher Scientific, Switzerland) supplemented with 1 mg/mL bovine serum albumin (BSA, 11020021, Gibco, Thermo Fisher Scientific) and 10 mM D-(+)-glucose (G5400, Sigma–Aldrich). The supernatant was then removed and resuspended three times with 5 mL of Neurobasal medium (21103-049) supplemented with 2% B27 (17504-044), 1% GlutaMAX (61965-026), and 1% penicillin streptomycin (15140-148; all from Thermo Fisher Scientific). The remaining solution was gently triturated to break up remaining tissue clumps. Glass 12 mm coverslips were activated with air plasma for 2 min (18 W PDC-32G; Harrick Plasma, USA) and immediately immersed in 0.1 mg/mL poly-D-lysine (PDL, P7280, Sigma-Aldrich) in PBS for 45 min. Subsequently, the coverslips were washed 3 times with PBS before transfer to 24-well plates (TPP, Faust, Switzerland). For neuronal cultures, the dissociated cells were plated onto the treated coverslips at the following densities: 1,000, 2,500, 5,000, 10,000, 20,000, and 50,000 cells/cm^2^. One day after cell seeding, the medium was replaced to remove any floating debris. Immediately afterwards, paper cultures were introduced to the experimental cultures designated for the co-culture condition.

For astrocyte paper cultures, instead of Neurobasal medium, cells were cultured in an astrocyte-promoting medium composed of DMEM (61965-026), 10% fetal bovine serum (FBS, 10270106) and 1% anti-anti (15240-062; all from Gibco, Thermo Fisher Scientific). Cells were plated at a density of 100,000 cells/cm^2^ on paper substrates, prepared as described in the subsequent section.

### Astrocyte feeder cultures

Standard Grade 2 cellulose filter papers of 8 μm particle retention and 190 μm thickness (Whatman, GE Healthcare, Switzerland) were cut into predefined shapes with a laser cutter (Speedy 300; Trotec Laser, Switzerland) before being air plasma-activated for 2 min in a polystyrene petri dish. The paper rings used for all experiments in 24-well plates had a 15.5 mm outer diameter and 6 mm inner diameter. After activation, the papers were transferred into a sterile 24-well plate under the laminar flow hood and immersed in one of the following surface functionalizing solutions: 10 μg/mL of laminin (L-2020, Sigma-Aldrich) in Neurobasal medium, 20 μg/mL of fibronectin (F1141, Sigma-Aldrich) in Neurobasal medium, or 200 μL of Matrigel (Corning Matrigel Basement Membrane Matrix; 354230, Chemie Brunschwig AG, Switzerland) diluted to 2 mg/mL in ice cold serum-free medium. After 45 min of incubation at room temperature, the papers were rinsed with supplemented Neurobasal medium and kept in Neurobasal medium and 2% penicillin streptomycin until seeding. Controls of 12 mm glass coverslips were similarly plasma-activated for 2 min and functionalized with 10 μg/mL of laminin in Neurobasal medium for 45 min.

### Co-culture platform

While paper cultures tend to float in the culture medium, they can move within the liquid and can come in contact with the substrate, in particular during manual manipulation of the well plates. To prevent direct contact, Parafilm (Parafilm, Faust) rings were attached to the side of the well plates used for experimental neuron cultures, as shown schematically in **Figure 2B**. Parafilm was first disinfected with 70% ethanol and then dried in the laminar flow hood. The Parafilm was then cut into bands and shaped into a ring before being placed with tweezers in a sterile 24-well plate 3 to 5 mm away from the bottom before cell seeding. After 1 week of culture, precursor astrocyte feeder cultures were transferred to the 1 day *in vitro* (DIV) experimental neuron cultures by submerging the paper in the medium using tweezers.

### Microcontact printing

To create structured networks of neurons, glass coverslips were patterned with cell-adhesive PDL on a cell-repulsive poly-L-lysine grafted polyethylene glycol (PLL-*g*-PEG, Surface Solutions, Switzerland) background reference surface (Ricoult et al., 2014) using microcontact printing. The protocol was adapted from (Ricoult et al., 2012). Polydimethylsiloxane (PDMS; Sylgard 184, Dow Corning) stamps were replicated from an SU-8 mold and submerged in ethanol overnight after curing to extract unpolymerized PDMS oligomers. Before stamping, the stamps were disinfected by ultrasonication in 70% ethanol for 15 min. The stamps were subsequently inked for 5 min with a solution of 10 μg/mL PDL and 25 μg/mL of Alexa Fluor 594 antibody as a fiducial marker (A-11032, Invitrogen, Thermo Fisher Scientific) in PBS. Before stamping on a plasma-activated coverslip, the stamp was rinsed for 5 s with PBS and then distilled water followed by drying with nitrogen. The patterned coverslip was then placed in a 24-well plate and submerged in a backfilling solution consisting of 20 μg/mL PLL-*g*-PEG in PBS for 40 min and finally rinsed with PBS.

### Immunocytochemistry

At 14 DIV, astrocytic feeder cultures were fixed in 4% paraformaldehyde (158127, Sigma Aldrich) at room temperature for 5 min then blocked for 1 h with 3% bovine serum albumin (BSA; AlbuMAX I Lipid-Rich BSA, 11020021, Thermo Fisher Scientific) and 0.1% Triton X-100 (T-8787, Sigma-Aldrich) in PBS. Samples were incubated overnight at 4°C in 3% BSA in PBS with the primary antibody chicken anti-glial fibrillary acidic protein (GFAP; 1:500; Sigma-Aldrich) to stain for glial cells. After three times washing for 10 min in 3% BSA in PBS, samples were incubated for 2 h in PBS with the secondary antibody goat anti-chicken IgG-Alexa Fluor 568 (1:500; Thermo Fisher Scientific) and the far-red nuclei marker DRAQ5 (1:1,000, Biolegend). Samples were washed again three times for 10 min in PBS and then mounted onto a glass WillCo dish (WillCo Wells B. V., Netherlands). Immunocytochemistry imaging was done on a LSM 510 confocal laser scanning microscope (CLSM; Carl Zeiss, Switzerland).

### Viability analysis

For the analysis of astrocyte viability on cellulose substrates, samples were assessed using the LIVE/DEAD Viability/Cytotoxicity Kit (2 μM Calcein AM and 4 μM Ethidium homodimer-1, L3224, Invitrogen, Thermo Fisher Scientific in PBS) at 7 DIV. Viability of the experimental neuron cultures was measured at 5 DIV using the live/dead stain combined with a nuclear counterstain. The cultures were washed with PBS after removal of the astrocyte paper cultures for the co-culture conditions followed by 12 min incubation with 250 μL of the live/dead stain. Next, the nuclear counterstain (250 μL of 1.62 μM Hoechst 33342, H1399, Invitrogen, Thermo Fisher Scientific in PBS) was added, incubated for 10 min, and subsequently washed with PBS.

For both astrocyte paper cultures and experimental neuron cultures, fluorescence microscopy pictures were taken of each sample at three randomized positions with an EM-CCD camera (Hamamatsu C9100-13, Hamamatsu Photonics K. K., Switzerland) on an Axio Observer Z1 inverted microscope equipped with a Colibri LED light source (Carl Zeiss, Switzerland) using μManager (Edelstein et al., 2014).

For image processing of astrocyte paper cultures, the number of live and dead cells was manually counted, and the three separate images per sample were summed. Due to slight auto-fluorescence of the cellulose paper at the excitation required to image the Hoechst stain, it was not possible to quantify the total number of cells with this method. Thus, simply the ratio of counted live cells to total number of cells was calculated.

For image processing of the experimental neuron cultures, the cells were counted using a custom Python script based on semi-automatic thresholding. The total number of cells and the number of dead cells were determined from the nuclear stain and dead stain, respectively. To calculate the viability per well, the number of live cells and dead cells of the three separate images were each summed and the ratio of live cells to total number of cells was calculated. The live stain was not used to count the number of live cells; instead the difference between total number of cells and dead cells was calculated. The density was calculated as number of live cells per imaged area.

### Neurite length analysis

The neurite length at 5 DIV was quantified using the ImageJ (Schindelin et al., 2015) neurite tracing plugin NeuriteTracer (Pool et al., 2008). The plugin was used to calculate the overall neurite length and number of cells for each fluorescence image previously used to calculate viability. The threshold for isolation of neurites and nuclei was determined manually for one image and then applied to all images of the same condition. The results of all images per culture were then summed. To account for variation between biological replicates, the fold change in neurite length was calculated separately for each biological replicate. In detail, for each density, the values of all co-culture and control samples were divided by the mean of the control samples.

### Activity analysis

Calcium imaging was performed at 14 DIV to measure the activity. Cultures were transduced at 7 DIV with a neuron-specific AAV GCaMP6f vector using a synapsin promoter (AAV1.Syn.GCaMP6s.WPRE.SV40; Penn Vector Core, USA; Chen et al., 2013). Half of the culture medium was replaced with virus stock solution diluted 1:1,000 with fresh culture medium.

At 14 DIV, calcium activity videos were recorded with an EM-CCD on an inverted fluorescence microscope. A video was taken at 3–5 positions per well for 2 min each with a frame rate of 32 Hz. The recorded data was analyzed using a custom Python program (available at neurons.ee.ethz.ch). In brief, images are first segmented into individual cells with a consistent user-set threshold adapting for minor image variation. Next, spikes are extracted from the calcium fluorescence intensity traces using wavelet-based spike detection. For all measured cultures, the number of detected spikes in all positions was summed up to calculate the total spike frequency. The mean spike frequency per cell was calculated by dividing the total spike frequency by the number of cells detected in all positions. Analogous to the neurite length analysis, the fold change in spike frequency was calculated separately for each biological replicate.

### Experimental design and statistical analyses

**Figure 1:**
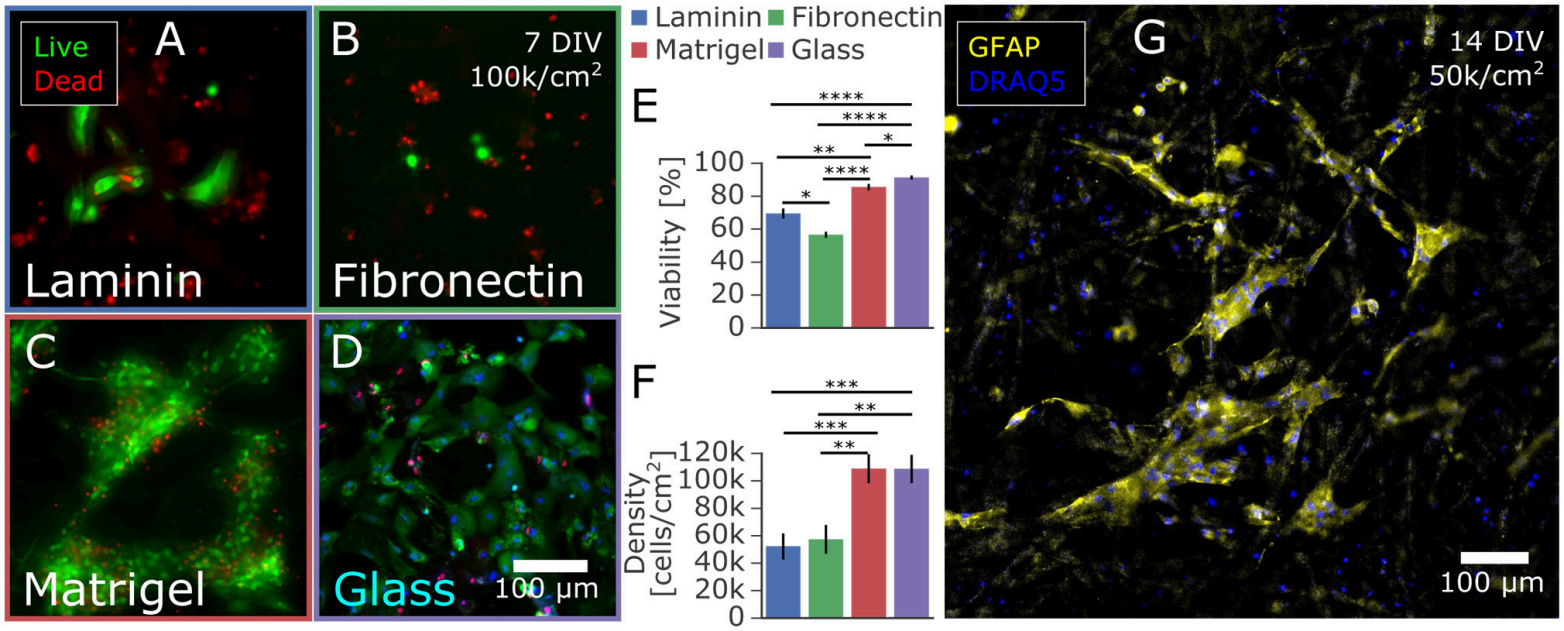
Astrocytes isolated from primary rat cortex and cultured on paper-based substrates. **(A–D)** Influence of extracellular matrix protein coatings on astrocyte proliferation and viability at 7 DIV. Astrocytes were seeded at 100,000 cells/cm^2^ on paper coated with **(A)** laminin, **(B)** fibronectin, or **(C)** Matrigel and on a control of **(D)** laminin-coated glass. After 7 days in culture with medium containing 10% FBS, the cells were stained with a live/dead viability assay. The cells on Matrigel-coated paper had the highest **(E)** viability and **(F)** number of adhered cells likely due to proliferation and/or adhesion properties in comparison to fibronectin and laminin coated paper. **(G)** Immunostaining with the astrocytic marker GFAP confirmed the astrocyte phenotype of the majority of rat cortex derived cells cultured for 14 days on Matrigel coated paper. Significance levels: ^*^p < 0.05; ^**^p < 0.005; ^***^p < 0.0005; ^****^p < 0.00005.

The effect of astrocytic co-culture was assessed on culture viability, neurite length and spiking activity of primary neuron cultures plated at densities ranging from 1,000 to 50,000 cells/cm^2^. To test the limit of co-culture, a lower limit of 1,000 cells/cm^2^ was chosen where only negligible survival and activity was expected (Brewer, 1995). The upper limit of 50,000 cells/cm^2^ was chosen as a commonly used density for *in vitro* experiments that reliably shows satisfactory viability and activity. All conditions were repeated with 2–3 biological replicates each consisting of 5 technical replicates. For each condition, an equal number of control experiments without astrocytic co-culture were performed. The effect of co-culture was compared to the control using the SciPy (Jones et al., 2001) implementation of the one-sided Mann-Whitney U test, which takes the non-normally distributed data into account. The reported values in Figures 1, **3**, and **6** are mean ± standard error, and in the boxplots in **Figures 4**, **5** the box corresponds to the first and third quartiles with the median. The ends of the whiskers are at the ends of the box, the median is indicated with a horizontal line in the box, and the maximum and minimum are at the ends of the whisker. Measurements 1.5 interquartile ranges below the first quartile or above the third quartile are classified as outliers and excluded. The reported significance levels are *p* < 0.05 (^*^), *p* < 0.005 (^**^), *p* < 0.0005 (^***^), and *p* < 0.00005 (^****^).

## Results

### Characterization of astrocytic paper-based culture

Whatman filter paper was previously shown to promote extensive neuronal growth into the cellulose fiber matrix, creating a dense 3D network especially within the Whatman Grade 2 membranes, which are characterized to have a porosity of 8 μm (Dermutz et al., 2017). We adapted the culture technique for astrocytic populations by seeding primary embryonic cortical cells onto laser-cut paper membrane rings and culturing them in astrocyte-promoting serum-based media. Since cellulose paper is a novel substrate for astrocytic culture, we first evaluated various surface functionalizations to optimize cell adhesion and viability. After sterilization and surface activation in a plasma cleaner, paper was submerged in a solution of laminin, fibronectin, or Matrigel to promote cell adhesion to these extracellular matrix proteins that adsorb to the paper. We compared viability and total cell count to a laminin-coated glass control at 7 days *in vitro* (Figures 1A–D). While astrocytes did develop on laminin-coated and fibronectin-coated paper substrates, Matrigel-infused substrates held over twice as many astrocytes and had comparable viability to cells on glass controls of around 90% (Figures 1E,F). Potential sources of the increased number of cells are improved initial adhesion, reduced cell death, and increased proliferation while in culture. All astrocyte feeder cultures further discussed were functionalized with Matrigel. The astrocytic phenotype was confirmed by immunostaining of the fixed samples targeting the astrocytic marker glial fibrillary acidic protein (GFAP) and counterstained with a nuclear marker DRAQ5, as reported in Figure 1G. Morphology and size of the GFAP-positive astrocytes were similar to those observed in other 3D substrates (East et al., 2009; Puschmann et al., 2013; Placone et al., 2015), exhibiting a lesser cell spread into three dimensions compared to the flattened, large spread of astrocytes grown on 2D glass. In addition, most of the cells in 3D exhibited a low level of GFAP expression characteristic of quiescent astrocytes *in vivo*, compared to the high level of GFAP expression typical to both 2D *in vitro* astrocytic cultures and the astrocytes *in vivo* that become reactive under pathological conditions (Pekny and Pekna, 2014; Hol and Pekny, 2015). Further investigations into 3D cell morphology were beyond the scope of the paper due to the complexity of characterizing dense 3D cultures. Paper membranes proved to be a robust, convenient platform to create astrocytic cultures that were likely more physiologically relevant than those on 2D substrates. The paper was also as easy to functionalize with adsorbed surface modifications as standard glass or tissue culture polystyrene.

### Effect of co-culture on viability

**Figure 2:**
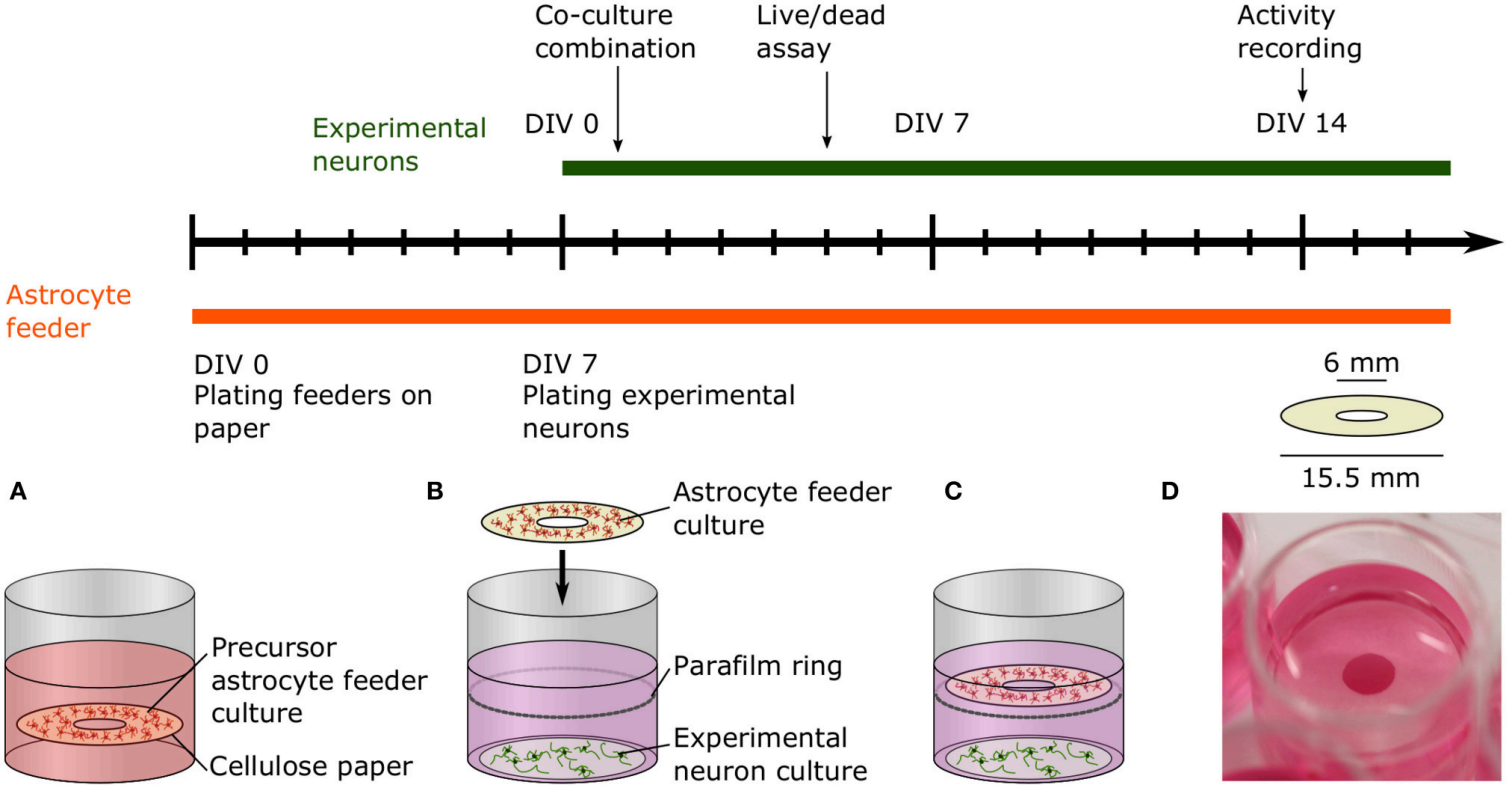
Timeline and schematic of the paper-based co-culture platform supporting low-density neuron cultures. **(A)** The precursor astrocyte feeders were cultured and proliferated on paper rings in medium supplemented with serum for 1 week before combination with the experimental neuron culture. **(B)** After a week, freshly dissociated neurons were seeded onto glass coverslips to create the experimental neuron cultures. After 24 h, the 8 DIV astrocyte feeder culture was added to the 1 DIV experimental culture. The paper ring was placed on top of a Parafilm ring submerged in the media to prevent direct contact of the two cultures. **(C)** Viability and neurite length of the experimental neuron culture was measured at 5 DIV with a live/dead assay. The experimental neuron culture activity was assessed at 14 DIV using calcium imaging. **(D)** Picture of the co-culture platform in a 24-well plate.

In low-density neuronal cultures, typically below 50,000 cells/cm^2^, individual cells and neurites can be easily resolved using light microscopy. Unfortunately, it is often difficult to sustain healthy cultures for more than a few days at such densities. Low viability of dissociated primary neurons has been commonly observed and reported, e.g., 65% for cortical neurons at 5,000 cells/cm^2^ after 4 days *in vitro* (Brewer, 1995). Despite the beneficial factors provided in specialized culture media, viability is limited because neurons additionally rely on paracrine trophic factors and cell-to-cell contact, which are both minimal at lower densities (Brewer, 1995). Furthermore, mono-cultures of only neurons lack all the essential functions of astrocytes that help to create a dynamic, supportive micro-environment. Co-culturing low-density neuronal cultures with a higher density culture has been shown to be helpful in mitigating these limitations (Kaech and Banker, 2006; Fath et al., 2009). Depicted in Figure 2, we devised a novel co-culture platform to support low-density neuronal networks with astrocytes cultured on paper. Astrocyte feeders are cultured separately in medium optimized for glial growth for 1 week then suspended above a 1 DIV neuronal experimental culture.

**Figure 3:**
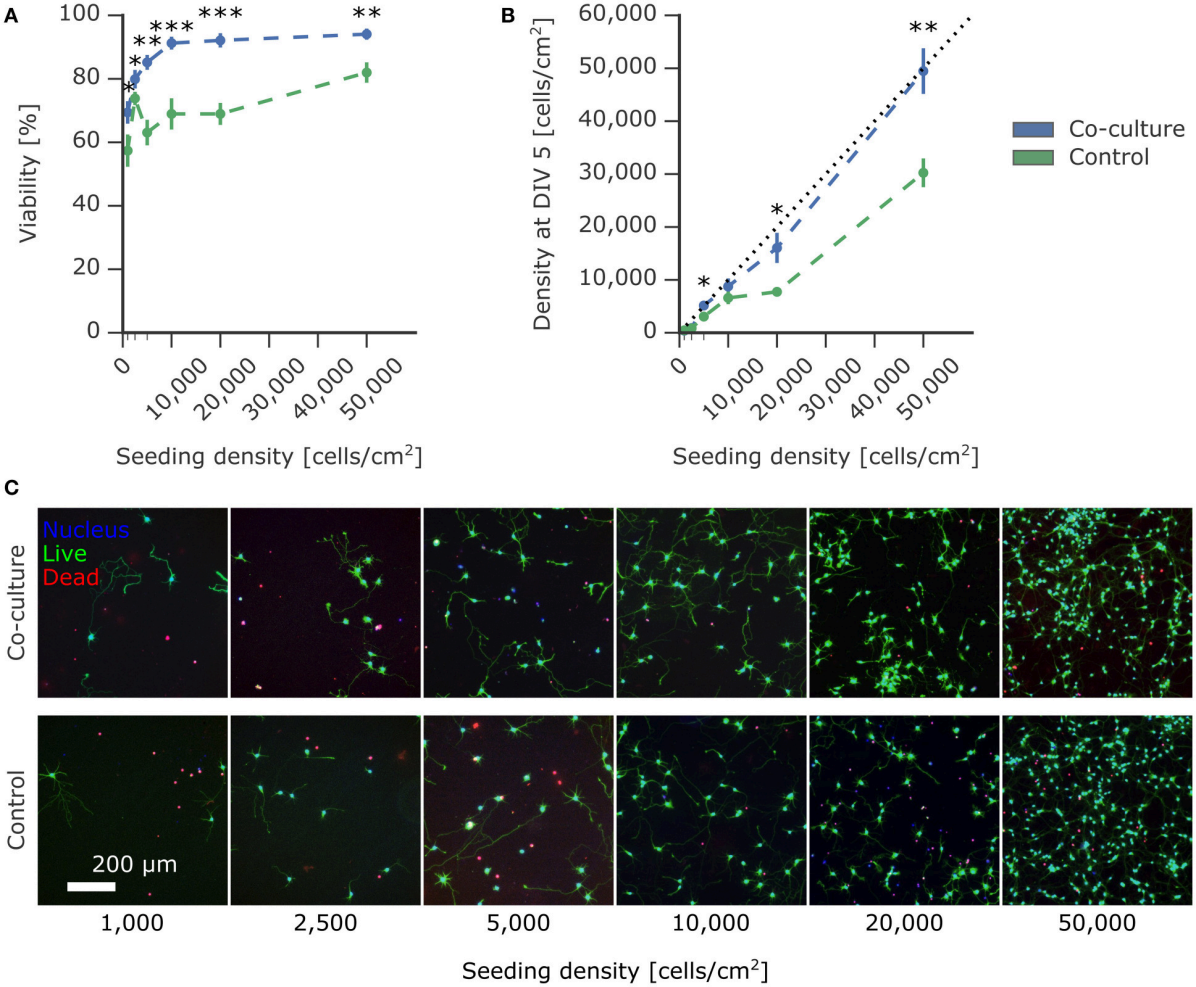
Effect of astrocyte co-culture on the viability of low-density neuron cultures. Primary rat cortical neurons were seeded on PDL-coated coverslips at densities ranging from 1,000 to 50,000 cells/cm^2^ with paper-based astrocyte co-culture and without co-culture as a control. The viability was measured using a live/dead viability assay at 5 DIV. **(A)** The viability of the experimental neuron culture supported by the co-culture was significantly improved compared to the control without co-culture. **(B)** The density of live experimental neurons on the coverslip was calculated using the live-dead assay images and compared to the seeding density. The co-culture condition showed minimal loss of cells compared to the control condition. **(C)** Representative images of the experimental neuron culture with and without co-culture at different seeding densities. Plotted values are mean ± standard error and significance was tested using Mann-Whitney U test with significance levels: ^*^ if *p* < 0.05; ^**^ if *p* < 0.005; ^***^ if *p* < 0.0005.

To quantify the effect of astrocytic co-culture on the survival of neuronal cultures, we cultured neurons at densities ranging from 50,000 cells/cm^2^ down to cultures of isolated cells at 1,000 cells/cm^2^. As a control, we repeated the same conditions but as mono-cultures without suspended astrocytic feeder cultures. In Figure 3A, there is a drastic improvement in viability across all densities compared to the control after 5 days *in vitro*, as measured using a live/dead assay. In the co-culture condition, viability steadily improved with increasing density until 10,000 cells/cm^2^, where it then plateaued at around 90%. Strikingly, viability for 1,000 cells/cm^2^ was still at 70%. In comparison, the control mono-culture viability mildly increased with density, albeit remaining at an overall lower viability without reaching a plateau within the range tested. Notably, at 10,000 cells/cm^2^, the control viability was only at 69% compared to 91% in the co-culture condition.

The quantification of the density of living cells at 5 DIV compared to the initial seeding density reported in Figure 3B further corroborates the supportive effect of astrocytic co-culture. In addition to the viability of the culture, this measure also took into account the commonly observed detachment of cells. Detached cells are predominantly dead, which can skew the viability obtained by a live/dead assay. Remarkably, the density of neurons supported by co-culture closely approached the theoretical limit (indicated with a dotted line), where the number of live cells at 5 DIV was equal to the number of seeded cells. Under control conditions, the resulting densities were significantly lower, with cell losses up to 50%.

In Figure 3A, the value at 2,500 cells/cm^2^ for the control viability was much higher than expected for this density. Unlike the viability measurement, the density of living cells is independent of dead cells. This density for the control condition at 2,500 cells/cm^2^ in Figure 3B was consistent with the other conditions, suggesting that the higher viability was caused by detachment of dead cells, commonly observed during washing steps in the viability assay protocol.

These experimental results demonstrate the effectiveness of suspended astrocyte paper cultures to improve survival of low-density neuronal cultures. This is, to the best of our knowledge, the first quantitative assessment of the effect of an astrocytic co-culture on viability at a wide range of densities.

### Effect of co-culture on neurite outgrowth

**Figure 4:**
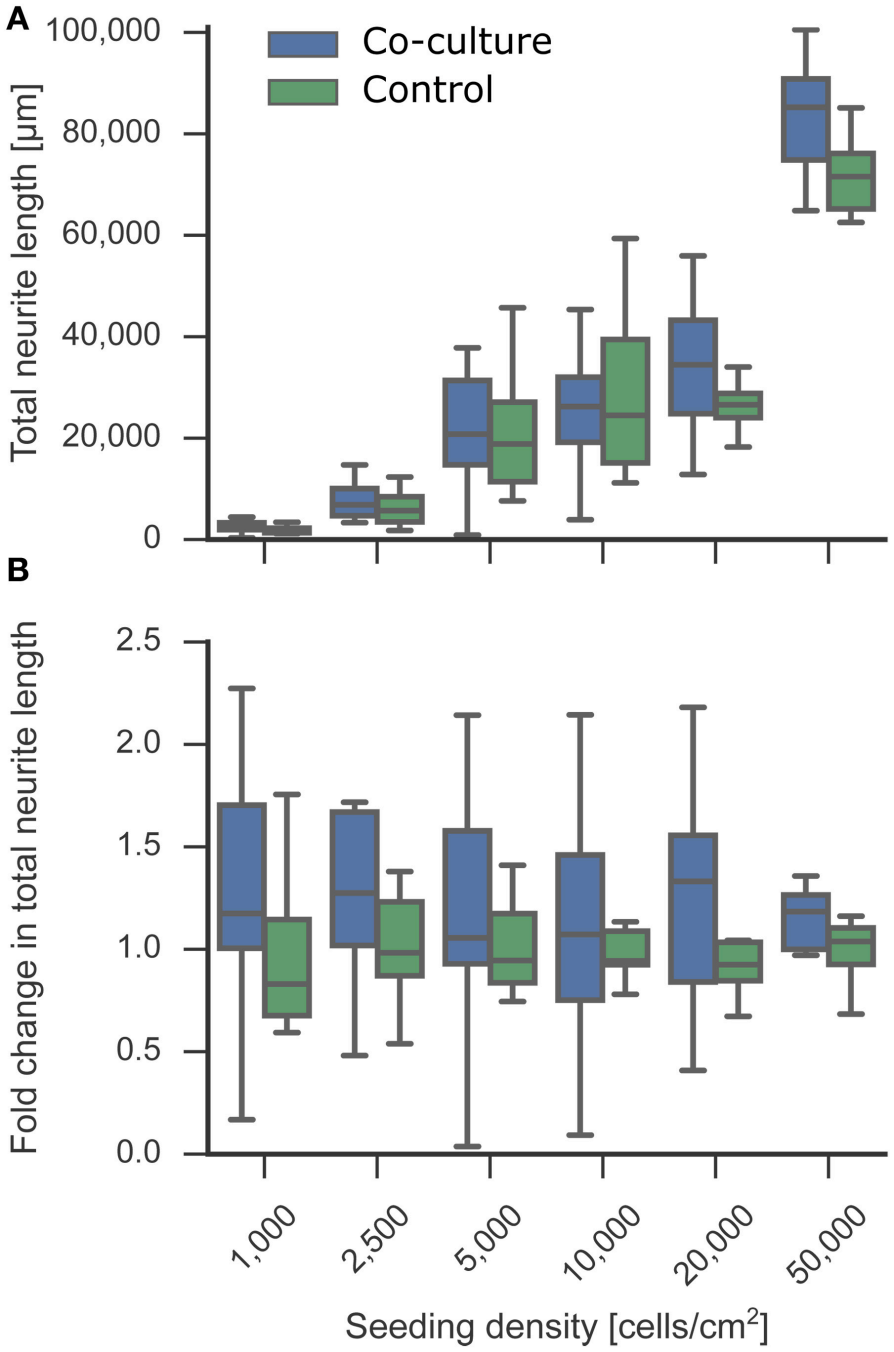
Effect of co-culture on neurite length as an indicator for neuronal development. The neurite length of the experimental neuron cultures in co-culture and control condition was measured at 5 DIV. Tracing and quantification was done using the ImageJ Plugin NeuriteTracer. **(A)** Comparison of neurite lengths of the experimental neuron cultures between co-culture and control conditions. **(B)** The fold change of the resulting total neurite length indicated a trend toward an increased neurite length in the co-culture condition compared to the mono-culture control. In the plot, the box corresponds to the first and third quartiles with the median. The median is indicated with a horizontal line in the box, and the maximum and minimum are at the ends of the whiskers. Measurements 1.5 interquartile ranges below the first quartile or above the third quartile are classified as outliers and excluded.

While general viability is critical for a successful *in vitro* bioassay, a more challenging factor to tune is the functionality of the cell type to match *in vivo* properties. Cell morphology and outgrowth relies heavily on components within the surrounding micro-environment, which is simplified in an *in vitro* system. We tested whether neurons exposed to the micro-environment provided by the astrocytic co-culture showed variable developmental neurite outgrowth compared to control mono-cultures. We measured the neurite length from the fluorescent staining of cell bodies and nuclei at 5 DIV with the ImageJ Plugin NeuriteTracer. As depicted in Figure 4A, the total neurite length increased with the seeding density, as can be expected. The supportive effect of co-culturing on neurite outgrowth is visible in Figure 4B, which shows fold change increase in neurite length under co-culture conditions relative to the control. While no significant increase was measured for the individual densities, an overall slight trend toward longer neurites was observed in the presence of the astrocyte paper cultures, although it is not possible to attribute the increase to a direct effect of the astrocytes on neurite length or to the overall increased viability in co-culture conditions. The extension of neurites, as characterized here, is an essential step toward forming a network of neurons, as more and longer neurites increase the chance of forming functional synaptic connections.

### Effect of co-culture on spiking activity

**Figure 5:**
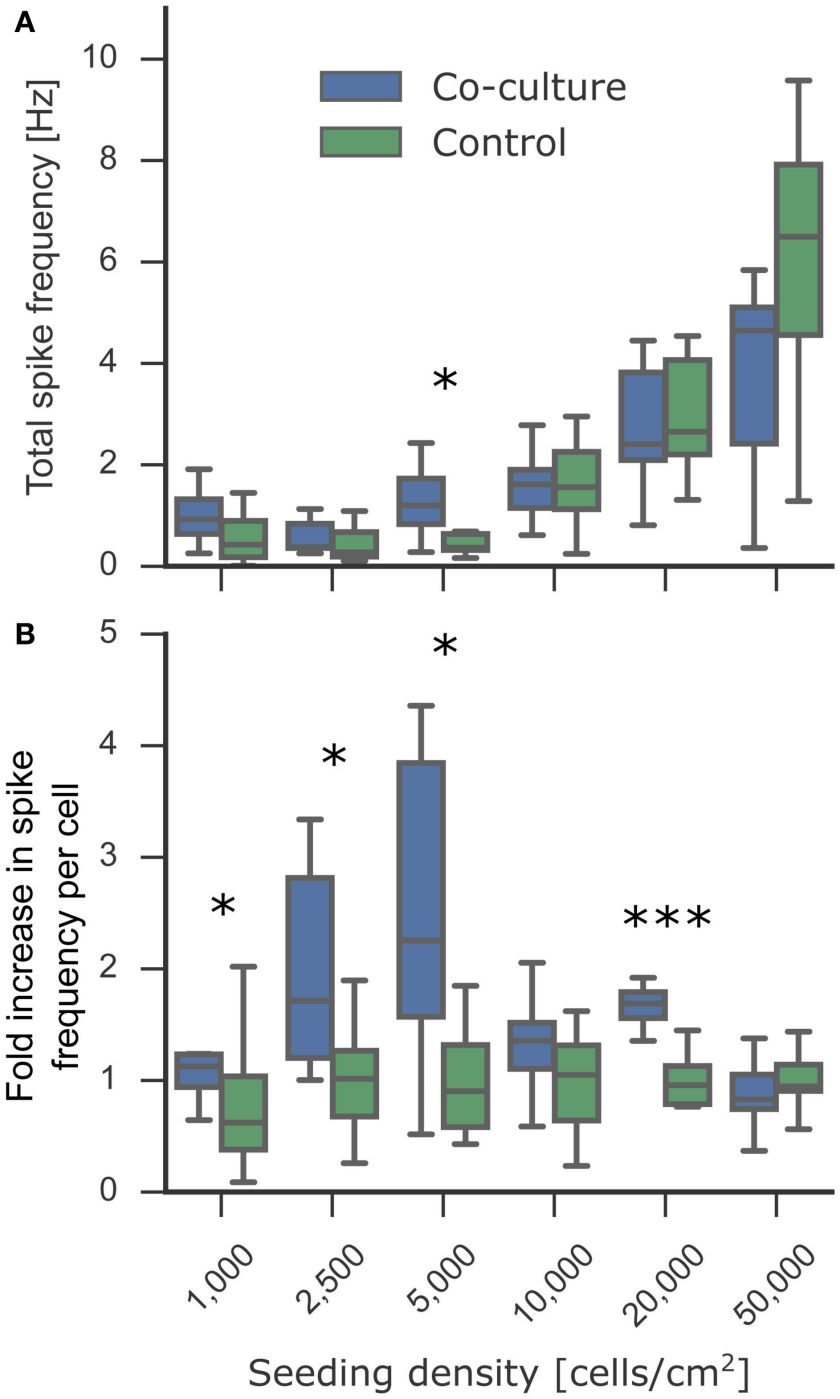
Effect of astrocyte co-culture on the spiking activity of low-density neuronal cultures. The cultures were transduced with the gene-encoded calcium indicator GCaMP6f at 7 DIV. At 14 DIV, the activity of the experimental neuronal cultures was measured using the fluorescent calcium indicators. To obtain the spiking activity, the calcium activity videos were segmented into individual cells followed by spike detection. **(A)** Spontaneous activity was detected in all cultures down to 1,000 cells/cm^2^. The culture-wide spike frequency increases progressively with the seeding density. **(B)** The fold increase in spike frequency per neuron between the co-cultured and control experimental neuron cultures showed an increase in spike frequency at low densities. In the plot, the box corresponds to the first and third quartiles with the median. The median is indicated with a horizontal line in the box, and the maximum and minimum are at the ends of the whiskers. Measurements 1.5 interquartile ranges below the first quartile or above the third quartile are classified as outliers and excluded. Statistical significance was tested using Mann-Whitney *U*-test with significance levels: ^*^ if *p* < 0.05; ^***^ if *p* < 0.0005.

*In vitro* cultures of neurons spontaneously exhibit rhythmic and synchronous electrophysiological activity similar to that in developing neurons in the nervous system (Chiappalone et al., 2006, 2007). This activity is indicative of connected neuronal networks. Low-density networks, however, often do not exhibit this activity because individual cells can be isolated and not well-connected to neighboring neurons. The previously established beneficial effect of co-culturing on viability and neurite outgrowth suggested a higher probability of interconnecting the sparsely distributed neurons at low densities. A connected network is required for synchronous activity, and an increased number of synapses could increase the frequency of spontaneous activity (Muramoto et al., 1993). Astrocytes also play an important role in the modulation of neuronal activity (Clarke and Barres, 2013), suggesting that co-culturing might improve electrophysiological function. Adding either astrocytes or astrocyte-conditioned medium improves the spontaneous spiking activity of neuronal cultures; however, glutamate-stimulated spiking activity is increased to a considerably higher extent only when co-culturing directly with astrocytes, indicating that dynamic interactions between neurons and astrocytes are likely necessary and that conditioned medium may only have a short-lived effect (Boehler et al., 2007). We measured 2 week old neuronal cultures in co-culture and mono-culture conditions and quantified the spike frequency obtained by calcium imaging (Figure 5A). We observed spontaneous activity in all cultures down to 1,000 cells/cm^2^, with the expected increase in total spike frequency at higher densities. Figure 5B demonstrates the supportive effect of astrocytic co-culturing on spiking activity based on the fold change in spike frequency compared to the control mono-culture. Co-culturing significantly increased spiking activity, most notably at all lower densities. This effect is less obvious in Figure 5A when comparing the total spike frequencies due to the high variability between biological replicates, which highlights the importance of analyzing the fold changes per biological replicate. These results may imply that the micro-environment generated by co-cultures improved neuronal signaling capabilities and increased spike frequency to more relevant levels that could be detected even in low-density cultures. This makes the suspended paper-based astrocyte co-cultures platform a promising technique for *in vitro* assays where activity is an especially critical measurement for determining the correct functionality compared to network activity *in vivo*.

### Versatility of co-culture platform

Engineering neuronal networks with well-defined topologies has sparked the community's interest for investigating the relationship between network structure and function. Patterned networks can be tailored to a specific research question with high fidelity while additionally reducing the high variability present in random, non-patterned networks. Common patterns used for network assays, such as grids and stripes, are notorious for requiring low cell densities to avoid cell clumping on the sparse pattern and thus suffer from low cell viability and lack of activity.

**Figure 6:**
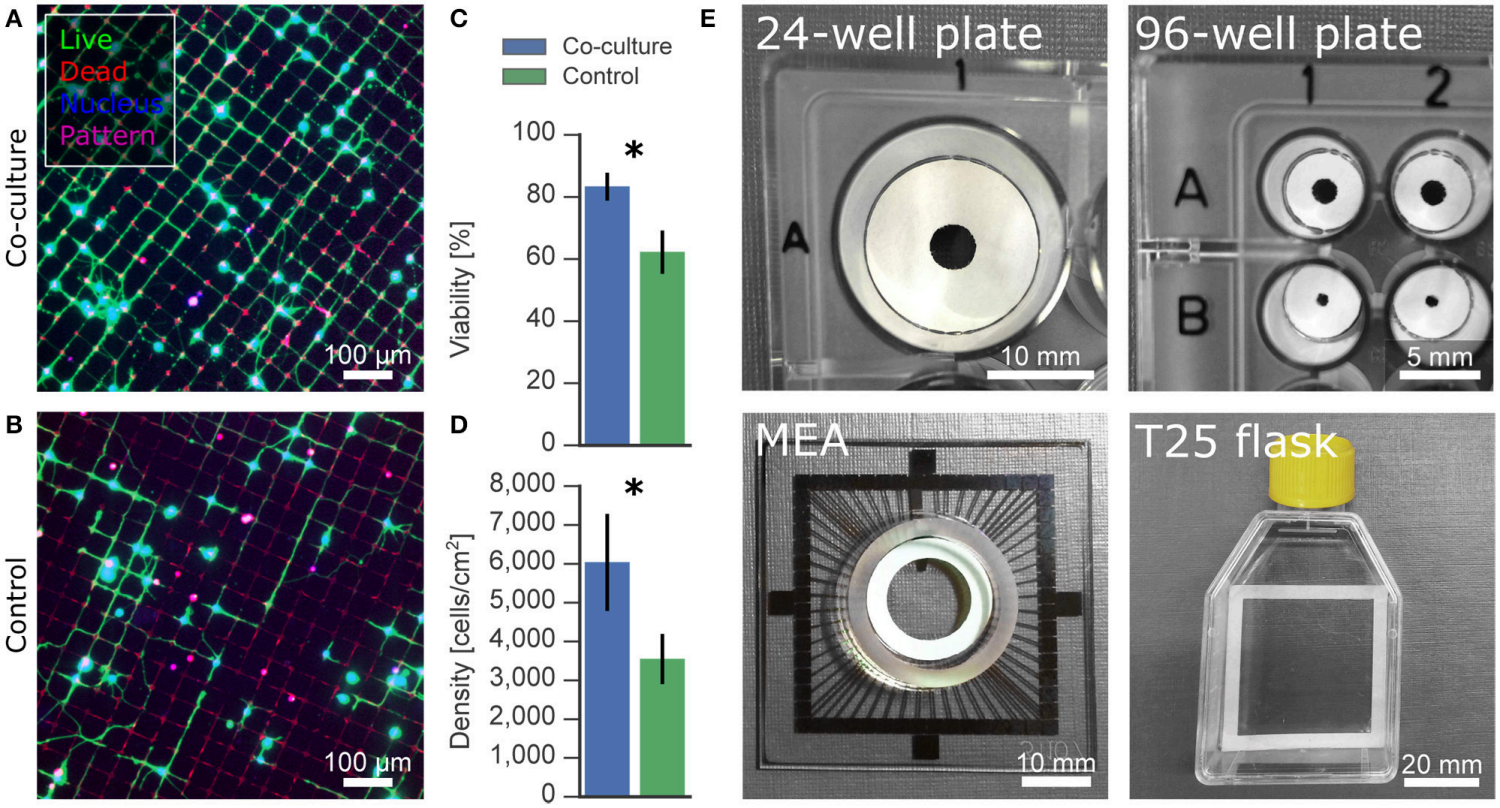
Applications and versatility of paper-based co-culture platform. **(A,B)** Effect of astrocytic co-culture on low-density neurons on microcontact-printed patterns. Patterned networks often require low densities to avoid clumping of neurons on the cell-adhesive regions, resulting in low viability. **(C,D)** Cell viability and density was improved under co-culture condition compared to control mono-cultures. **(E)** Paper-based culture can be structured to fit any culture vessel, such as well plates, multielectrode arrays and flasks, for versatile applications. Significance levels: ^*^p < 0.05.

Microcontact printing was used to generate grid patterns of cell-adhesive poly-D-lysine on a cell-repulsive PLL-*g*-PEG background. We seeded cells at 10,000 cells/cm^2^, which resulted in distinct networks without cell clumping but with poor viability, as evident in Figure 6A. Applying the co-culture platform presented here significantly improved the viability from 62% to 84% at 5 DIV and almost doubled the density of live cells from 3,500 cells/cm^2^ to 6,700 cells/cm^2^ (Figures 6B–D). In addition to our co-culture substrates optimized for 24-well plates, the shape of the cellulose substrate can easily be customized to a wide range of culture vessels, including but not limited to a 96-well plate, a standard commercial multielectrode array, and a T25 culture flask (Figure 6E).

## Discussion

The need for more physiologically relevant *in vitro* bioassays generates demand for a neuron-astrocyte co-culture platform that is well-characterized yet simple to use. While existing methods have been essential for advancing knowledge of neuron-astrocyte interactions, they can be cumbersome and expensive. Furthermore, they are frequently lacking general characterization, in particular in the context of low-density cultures.

### Characterization of astrocytic paper-based culture

As a well-characterized cell culture substrate, glass coverslips have been used in co-culture systems by suspending them above or next to the experimental culture (Park et al., 2001; Jones et al., 2011). When suspended, glass presents a physical barrier that prevents the diffusion of essential gasses and nutrients. Culturing on the side creates a non-uniform diffusion gradient of trophic factors across the experimental culture. Cellulose paper has been established as a cell culture platform that is permeable and does not act as a complete physical barrier (Derda et al., 2009; Dermutz et al., 2017). The 3D fiber matrix also more closely resembles the physiological conditions in tissues. Furthermore, the sturdy paper can easily be handled for addition and removal at any point during the experiment. It can be shaped according to the form and dimensions of the culture vessel. These properties make paper an ideal candidate for a neuron-astrocyte co-culture platform. Bacteria and plant-based cellulose is a commonly used biomaterial (Vandamme et al., 1998), in particular as a surface coating (Missoum et al., 2013). Early studies in the 70s aimed to bridge lesions in the spinal cord with astrocyte-infused paper implants (Eng et al., 1986). In more recent work, cellulose in its paper form has been rediscovered as a convenient substrate particularly for microfluidic diagnostics and *in vitro* cell-based assays. Our approach presented here establishes paper as a substrate for astrocytic culture with the aim of improving viability and development of low-density neuronal cultures via co-culture.

As the first step of the optimization process, the evaluation of different surface functionalizations showed a drastic difference in performance as a substrate for astrocyte culture, as evident in Figure 1. After 7 days in culture, Matrigel-coated paper contained twice as many cells than fibronectin and laminin-coated substrates in addition to significantly improved cell viability. We attribute the increased number of cells on Matrigel, a reconstituted basement membrane mixture of adhesion-promoting extracellular matrix proteins, to a combination of better adhesion after plating, survival and proliferation (Kleinman and Martin, 2005; Hughes et al., 2010; Levy et al., 2014). Notably, cellulose was compatible with standard coating methods for glass and plastic substrates. As a consequence, surface functionalization with adhesion-promoting proteins did not require significant adaptation.

While the viability and number of cells at 7 DIV of Matrigel-coated paper was comparable to the laminin-coated glass control, the cell morphology had characteristic differences between the 2D and 3D substrates, similar to as previously reported in the literature (East et al., 2009; Puschmann et al., 2013; Placone et al., 2015). Cells cultured on the cellulose fiber matrix thrived and formed a dense 3D network of small, stellate shaped cells compared to the large, flat cell structure typical of astrocytes on 2D glass. Immunostaining proved that the majority of cells stained specifically for GFAP, an astrocyte specific marker. In conclusion, we established Matrigel-coated paper as an astrocyte culture substrate ideally suited for co-culturing because of its permeability, ease-of-use, and 3D structure.

Designed specifically for co-culturing in 24-well plates, the ring shape of the paper allowed for convenient visual inspection through the center hole without removal of the paper, while maximizing the surface area available for astrocytes. Due to the neutral buoyancy of paper, the astrocyte cultures floated within the culture medium. However, as a preventative measure against mechanical damage to the underlying culture, in particular during transportation, a Parafilm spacer ring was added below the paper to ensure separation. Parafilm is inert and biocompatible (Yoo and Nam, 2012) and did not interfere with the cell culture. As outlined in the experimental timeline in Figure 2, precursor astrocyte cultures were matured for 1 week prior to combination with the 1 DIV experimental neuronal cultures. At 1 week, astrocytes populated the Matrigel-paper matrix at a sufficient density to be able to provide support to the low-density neuronal cultures. The supportive effect on cell viability was first assessed after 5 DIV, after the plateauing of initial cell death common to primary neuronal cultures. The time point was also used to analyze neurite length, as there was sufficient neurite growth at 5 DIV without dense neurite overgrowth that obscures quantification. Additional experimental cultures were developed for 14 DIV with a gene-encoded calcium indicator to quantify the effect of co-culturing on electrophysiological activity. At this time point, neurons have formed well-connected networks, which can exhibit spontaneous activity detectable by calcium imaging.

### Effect of co-culture on viability

In addition to enhancing general biological relevance, astrocyte co-cultures for *in vitro* assays are critical for improving survival and functionality of low-density cultures, including those needed for the bottom-up neuroscientific approach of building well-defined neuronal networks. While the positive effects of astrocytes on neuron cultures are broadly accepted, their influence on viability of low-density neuronal networks has not been sufficiently examined.

Here, we show that continuous support from astrocytes achieved a remarkably high viability of above 90% for densities down to 10,000 cells/cm^2^. Even for isolated cells at a density of 1,000 cells/cm^2^, the effect of co-culturing was significant with a viability of 69%. In contrast, mono-cultures required ten times more cells to reach this same viability. Overall, co-culturing dramatically shifted the viability curve toward lower densities, achieving high viability at densities where single cells and neurites can easily be resolved and analyzed. This enables the targeting of single cells in experiments that were previously hindered by prohibitively low viability. The comparison of our results with alternative methods is difficult, as quantification across seeding densities is lacking. Nevertheless, the limited published viability measurements are comparable to our analysis (Brewer and Cotman, 1989; Lucius and Mentlein, 1995; Pyka et al., 2011).

The standard live/dead assay tends to overestimate viability because it is unable to measure the unavoidable fraction of cells that die and detach during the initial phase after cell seeding. A more complete quantification is to measure the density of live cells compared to the initial seeding density. We observed a substantial loss of cells in mono-cultures measured after 5 days. Mono-cultures seeded at 50,000 cells/cm^2^ resulted in a density of only 30,000 cells/cm^2^, while more than half of the cells were lost at a seeding density of 20,000 cells/cm^2^. In comparison, the seeding densities and the actual densities after 5 DIV in co-cultures were closely matched, with greater than 80% efficiency for densities of 5,000–50,000 cells/cm^2^. We attribute this higher efficiency to an improved viability induced by co-culturing, which reduced the amount of detaching dead cells. Unlike conditioned medium, this platform provides a convenient way to give continuous support of potentially short-lived factors without the need of maintaining separate cultures to produce the medium and of frequently exchanging the medium.

Viability is influenced by both the surrounding micro-environment and by direct cell-cell contact (Brewer et al., 1993). The improved viability of more than 90% at 10,000 cells/cm^2^ and above indicates that the physically separate co-culture can provide the appropriate micro-environment for enhanced cell survival. The change in viability below 10,000 cells/cm^2^ shows the point where supporting lower density cultures with secreted factors alone is not sufficient anymore, suggesting that direct cell-cell interactions are likely the limiting factor. It remains unclear to what degree reciprocal communication has an effect on compartmentalized neurons and astrocytes *in vitro*, particularly since cultures lack the organized, close proximity of astrocytes at synapses that is normal within the brain. Since gliotransmission and neurotransmission are integrally coupled functions at the synapse, it is possible that only slower, long-range communication between cell types plays a significant role in these separated cultures. While direct culturing of astrocytes with neurons offers the added effect of cell-to-cell contact-mediated effects on viability, it introduces an additional layer of complexity from the multitude of inevitable interactions between the two cell populations. Thus, many *in vitro* assays require separation of cell types to obtain clear experimental cause and effect.

### Effect of co-culture on neurite outgrowth

Across all densities, neurons developed rapidly with extensive neurite outgrowth in both co-cultures and mono-cultures. The astrocyte co-cultures were added to 1 DIV neuronal cultures and were thus present during the critical period of neurite development following initial cell adhesion. Because astrocytes have been shown to positively influence neurite outgrowth (Smith et al., 1990; Filous et al., 2010), we measured the length of neurites at 5 DIV to characterize the extent of the astrocytic effect *in vitro*.

We observed a slight trend toward increased neurite length in co-culture conditions compared to control. The measured increase in neurite length could have resulted from an increase either in total number of neurites or in longer neurites. However, with the measurement method used, it was not possible to distinguish between the two cases. While it is also difficult to differentiate between the direct effect of astrocytes on the neurite outgrowth and the indirect effect caused by an overall higher viability, a denser network of neurites increases the chance of forming synaptic connections. This effect is particularly important in low-density cultures where individual cells are spaced far apart and are less likely to make connections and develop into active networks.

### Effect of co-culture on spiking activity

Studies using low-density neuronal cultures rarely investigate electrophysiological activity because of the difficulty to keep the cultures alive long-term until synapse maturation and onset of spiking activity. Neuromodulation of spiking properties has been an important experimental target for *in vitro* assays. Our results demonstrated the beneficial effect of co-culturing on neurite outgrowth, a crucial step toward electrophysiologically active networks. We investigated how co-culturing low-density neuronal networks with astrocytes for 2 weeks influenced their development into functionally connected and active networks. Astrocyte cultures were removed during the activity measurement to isolate the effect of co-culture on the development of the neuronal network while excluding any real-time regulatory effects. Cultures exhibited spontaneous spiking even at a very low density of 1,000 cells/cm^2^, with a strong trend toward greater total spike frequencies in co-culture conditions compared to control. When comparing the average spike frequency per cell, co-culturing resulted in a significantly greater spike frequency at lower densities. Notably, with our platform, the astrocyte co-culture could alternatively remain during measurements to investigate specific neuromodulatory effects of astrocytes.

The use of a gene-encoded calcium indicator made it feasible to repeat every condition at least five times per biological replicate to minimize the variability caused by cell population heterogeneity. At such low densities, where only few cells can be measured, a sufficient sampling size is particularly necessary for adequate quantification. Nevertheless, a more in depth study of electrophysiological properties could be achieved using multielectrode arrays (MEAs), which are better for probing temporal dynamics of networks but often with the tradeoff of a lower sample size (Ito et al., 2010; Geissler and Faissner, 2012). In a previous study, the minimum density for spontaneous activity was found to be 25,000 cells/cm^2^ for cortical neurons grown on a MEA in astrocytic conditioned medium (Ito et al., 2010). Even without co-culturing, we observed spiking activity below 25,000 cells/cm^2^, possibly because of the greater number of cells measured separately on account of improved spatial resolution with calcium imaging.

### Versatility of co-culture platform

Having characterized the supportive effect of our co-culture platform, we explored the potential effect on patterned networks, a popular application deterred by low viability and activity at low densities. The supportive effect of co-culturing in random cultures was also observed on an interconnected grid of neurons. Co-cultures seeded at a density of 10,000 cells/cm^2^ reached a viability of 83% and a density of living cells of 6,000 cells/cm^2^ after 5 DIV. In comparison, the control cultures measured significantly lower with a viability of 69% and a density of 3,550 cells/cm^2^, only a third of the initial seeding density and half of the co-culture. Compared to random, non-patterned cultures, the absolute viability and density values measured after 5 DIV were lower for neurons on grid patterns. The overall area for cells to adhere was reduced on the grid pattern, where the nodes are just big enough for one cell body and the thin lines can only support a few neurites. Cells that do not initially land on the pattern fail to adhere on the non-fouling background, which results in a lower cell density and thereby also a lower viability. In conclusion, these preliminary results with patterned networks of neurons show the potential of this co-culture platform for improving the cell culture conditions in bottom-up neuroscience.

While we have optimized the co-culture platform toward improving neuronal survival with astrocytic support, it is not limited to this application. Based on the experimental paradigm, the platform could easily be adapted for different cell types and different setups. The feeder cultures and experimental cultures can initially be kept separate, so there is complete flexibility for variations in media conditions, patterning techniques, patterns, and cell densities. Due to the ease of transferring the paper substrate, the combination and removal, if required, of the two separate cultures is simple and robust at any chosen time point. The co-culture can also be added at multiple time points, if necessary. Furthermore, the cellulose substrate is commercially available as Whatman filter paper in different forms, porosities, and thicknesses. The substrate can conveniently be adapted to fit any culture vessel by folding, rolling, or cutting. While laser cutting produces a large amount of substrate quickly, it is also possible to use a scalpel, paper puncher, and scissors. Standard surface chemistry modifications can additionally be applied to the cellulose membrane depending on what is optimal for a specific cell type. Overall, our platform is a robust, versatile approach that can easily be customized toward different experimental aims.

## Conclusion

Here, we have introduced a reliable, robust technique to conveniently co-culture astrocytes with neuronal cultures that is simple and inexpensive. The inclusion of astrocytes enhances *in vitro* bio-assays due to the essential neuron-astrocyte interactions that improve neuronal survival and function. We quantified this supportive effect on low-density neuronal cultures at a wide range of cell seeding densities, from isolated cells to well-connected networks. In co-culture conditions, there was an increase in viability and spiking activity, in particular at low densities. Using a paper-based substrate additionally makes the platform customizable to different applications that will further enable research in bottom-up neuroscience and drug development.

## Author contributions

MA, GT-S, and JV designed the research project. MA and GT-S performed and analyzed all the experiments. AJ performed preliminary experiments. MA, CB, and MS developed the calcium imaging software. HH produced the mold for microcontact printing. SW and CF gave critical input and discussion. MA and GT-S wrote the paper. All co-authors reviewed the paper.

### Conflict of interest statement

The authors declare that the research was conducted in the absence of any commercial or financial relationships that could be construed as a potential conflict of interest.
